# Inpatient Outcomes of Acute Pancreatitis Among Patients With Systemic Lupus Erythematosus: A Nationwide Analysis

**DOI:** 10.7759/cureus.16349

**Published:** 2021-07-13

**Authors:** Daniel Rim, Alexander Kaye, Catherine Choi, Sushil Ahlawat

**Affiliations:** 1 Internal Medicine, Rutgers University, Newark, USA; 2 Gastroenterology and Hepatology, Rutgers University, Newark, USA

**Keywords:** acute pancreatitis, systemic lupus erythematosus, outcomes, mortality, characteristics

## Abstract

Objectives

This study explores the characteristics and outcomes, including inpatient mortality, length of stay, and pancreatitis complications in patients hospitalized with acute pancreatitis (AP) with coexisting systemic lupus erythematosus (SLE).

Methods

Patients hospitalized with AP from the National Inpatient Sample from 2014 were selected. Patient characteristics and outcomes of AP were compared between the groups with and without SLE. Age, sex, race, Elixhauser Comorbidity Index (ECI), and etiologies of pancreatitis were measured. The outcomes of interest were inpatient mortality, length of stay, and complications, including respiratory failure, acute renal failure, myocardial infarction, hypotensive shock, sepsis, stroke, and ileus. Chi-squared tests and independent t-tests were used to compare proportions and means, respectively. Multivariate logistic regression analysis was performed to determine if SLE is an independent predictor for the outcomes, adjusting for age, sex, race, ECI, and etiologies of pancreatitis.

Results

Among 434,280 AP patients identified in the study, 3,015 patients had SLE. Among patients hospitalized with AP, those with SLE were younger, more likely to be female, more likely to be non-White, had higher ECI, and stayed longer in the hospital. Patients without SLE were more likely to have a history of cholelithiasis, alcohol abuse, and hypertriglyceridemia. AP patients presenting with SLE were at higher risk for respiratory failure, acute renal failure, hypotensive shock, stroke, and sepsis. Higher inpatient mortality was also associated with coexisting SLE.

Conclusions

Patients admitted for AP with SLE have worse outcomes compared to those without SLE. Understanding the potential effects of SLE on AP and optimizing patient care in this population accordingly may improve the quality of care and outcomes.

## Introduction

Acute pancreatitis (AP) is a common pathology with many etiologies. AP has an estimated incidence of 300 to 500 cases per million patients per year in the Western world and accounts for about 220,000 hospital admissions per year in the United States [1,2]. The cause of AP can be identified in about 75% to 85% of cases [3]. The most common causes of AP include obstructing gallstone (in approximately 38% of cases) and significant alcohol use (in approximately 36% of cases) [3]. There are many other less common causes of AP, including systemic lupus erythematosus (SLE).

SLE is an autoimmune disease that has a wide range of presentations. The prevalence of SLE differs greatly depending on geographic location, but it is found most commonly in North America, with a prevalence of 241 out of every 100,000 people [4]. SLE is more common in women, with a ratio of women to men estimated between 1.2:1 to 15:1, as well as most often found in patients of black ethnicity [4]. SLE-associated pancreatitis is diagnosed in SLE patients when no other etiology is obvious. SLE was first documented as a cause of pancreatitis in 1939 [5,6]. The approximate incidence of SLE-associated pancreatitis is between 0.4 and 1.1 per 1000 patients with SLE [7]. The mortality rate of SLE-associated pancreatitis has been observed to be as high as 27% [8]. 

While there is a high mortality associated with SLE-associated pancreatitis, research is lacking on how coexisting SLE affects AP’s characteristics and clinical outcomes, regardless of AP etiology. Despite a case series comparing AP patients with and without SLE showing higher mortality in the SLE group, more robust studies did not follow [9]. This article addresses this issue by exploring and comparing the characteristics, and clinical outcomes in adult patients admitted for AP with coexisting SLE compared to those diagnosed with AP but without SLE. This article was previously presented as a meeting abstract at the ACG 2020 Annual Scientific Meeting in October 2020.

## Materials and methods

This study is a retrospective cohort study that involves adult patients (age 18 and above) who were hospitalized with AP in the United States in 2014 using data from the National Inpatient Sample (NIS), Healthcare Cost and Utilization Project (HCUP), and the Agency for Healthcare Research and Quality, which is known as the largest all-payer inpatient database in the United States [10]. Diagnoses were identified by using The International Classification of Diseases, Ninth Edition Revision, Clinical Modification (ICD-9 CM) codes.

Patients were then divided into two groups: those with and without the diagnosis of SLE. Patient demographics and characteristics including age, sex, race, Elixhauser Comorbidity Index (ECI), and presence of other etiologies of pancreatitis such as cholelithiasis, alcohol abuse/dependence, hypertriglyceridemia, obstruction of the bile duct, and hypercalcemia were compared between the groups. The use of endoscopic retrograde cholangiopancreatography as an etiology of AP was initially considered but omitted due to the small sample size in the study. ECI is a system of measuring 29 comorbid conditions to assess their impact on the outcomes of patients [11,12]. The clinical outcomes of AP including inpatient mortality, length of stay, respiratory failure, acute renal failure, hypotensive shock, ileus, mechanical ventilation (MV), myocardial infarction (MI), stroke, sepsis, pulmonary embolism (PE), and portal vein thrombosis were collected and compared between the groups. Other potential outcomes such as deep vein thrombosis and parenteral nutrition were not included in the study due to small sample sizes.

Statistical Package for the Social Sciences (SPSS), version 26.0 (IBM Corp, Armonk, NY), was used for all statistical analyses. Chi-squared tests and independent t-tests were used to compare proportions and means, respectively. Statistical analyses performed in this study were two-tailed, and a p-value less than 0.05 was considered statistically significant. Continuous variables were reported as means ± standard deviation (SD), whereas categorical variables were reported as numbers (N) and percentages (%). Multivariate logistic regression analysis was performed to determine if SLE is an independent predictor for the clinical outcomes, adjusting for age, sex, race, ECI, and etiologies of pancreatitis, including cholelithiasis, alcohol abuse/dependence, hypertriglyceridemia, obstruction of the bile duct, and hypercalcemia.

## Results

Among 434,280 patients with AP identified in the study, 3,015 patients had SLE. Patient demographics and characteristics are demonstrated in Table 1. Among patients hospitalized with AP, those with SLE were younger (48.0 vs 53.5, p < 0.05), more likely to be female (89.1% vs. 51.6%, p < 0.05), less likely to be White (48.0% vs. 65.7%, p < 0.05), and had higher ECI (7.5 vs. 5.7, p < 0.05). Patients without SLE were more likely to have history of cholelithiasis (21.5% vs. 14.8%, p < 0.05), alcohol abuse/dependence (12.7% vs. 4.8%, p < 0.05), and hypertriglyceridemia (4.7% vs. 2.2%, p < 0.05). There were no statistically significant differences in prevalence of obstruction of the bile duct (p = 0.051) and hypercalcemia (p = 0.675) between the groups.

**Table 1:** Demographics and characteristics among AP patients with and without SLE AP, acute pancreatitis; SLE, systemic lupus erythematosus; ECI, Elixhauser Comorbidity Index; SD, standard deviation

Variable	AP with SLE	AP without SLE	p-value
N = 434,280	N = 3,015	N = 431,265	
Patient age, mean (SD)	48.0 (14.7)	53.5 (17.5)	<0.05
Sex, N (%)			<0.05
Female	2,685 (89.1%)	222,255 (51.6%)	
Male	330 (10.9%)	208,865 (48.4%)	
Race, N (%)			<0.05
White	1,400 (48.0%)	269,340 (65.7%)	
Black	1,025 (35.2%)	63,290 (15.4%)	
Hispanic	350 (12.0%)	51,970 (12.7%)	
Asian or Pacific Islander	60 (2.1%)	9,490 (2.3%)	
Native American	15 (0.5%)	3,875 (0.9%)	
Other	65 (2.2%)	12,240 (3.0%)	
ECI, mean (SD)	7.5 (10.2)	5.7 (9.3)	<0.05
Etiology of AP, N (%)			
Cholelithiasis	445 (14.8%)	92,715 (21.5%)	<0.05
Alcohol abuse/dependence	145 (4.8%)	54,585 (12.7%)	<0.05
Hypertriglyceridemia	65 (2.2%)	20,285 (4.7%)	<0.05
Obstruction of the bile duct	80 (2.7%)	9,215 (2.1%)	0.051
Hypercalcemia	30 (1.0%)	3,975 (0.9%)	0.675

Table 2 compares clinical outcomes of AP in groups with and without SLE. Patients with coexisting SLE had higher prevalence of respiratory failure (7.1% vs. 5.2%, p < 0.05), acute renal failure (16.9% vs. 13.3%, p < 0.05), hypotensive shock (8.5% vs. 5.4%, p < 0.05), stroke (0.8% vs. 0.4%, p < 0.05), and sepsis (10.0% vs. 7.6%, p < 0.05). SLE patients also stayed longer in the hospital (6.9 vs. 5.7 in days, p < 0.05) and had higher inpatient mortality (3.3% vs. 2.0%, p < 0.05). Interestingly, patients without SLE had higher prevalence of ileus (3.8% vs. 3.0%, p < 0.05). There was no statistically significant difference in the prevalence of MI (p = 0.81). Due to small sample sizes, further analyses of MV, PE, and portal vein thrombosis were not performed.

**Table 2:** Clinical outcomes among AP patients with and without SLE Values are reported as numbers (%) unless specified otherwise; AP, acute pancreatitis; SLE, systemic lupus erythematosus; MI, myocardial infarction; SD, standard deviation

Outcome	AP with SLE	AP without SLE	p-value
N = 434,280	N = 3,015	N = 431,265	
Respiratory failure	215 (7.1%)	22,325 (5.2%)	<0.05
Acute renal failure	510 (16.9%)	57,454 (13.3%)	<0.05
Hypotensive shock	255 (8.5%)	23,165 (5.4%)	<0.05
Ileus	90 (3.0%)	16,525 (3.8%)	<0.05
MI	50 (1.7%)	7,395 (1.7%)	0.81
Stroke	25 (0.8%)	1,875 (0.4%)	<0.05
Sepsis	300 (10.0%)	32,985 (7.6%)	<0.05
Mechanical ventilation	0 (0%)	15 (0%)	
Pulmonary embolism	0 (0%)	0 (0%)	
Portal vein thrombosis	0 (0%)	2,380 (0.6%)	
Length of stay in days, mean (SD)	6.9 (8.1)	5.7 (7.9)	<0.05
Inpatient mortality	100 (3.3%)	8,715 (2.0%)	<0.05

As shown in Table 3, after adjusting for age, sex, ECI, and etiologies of pancreatitis including cholelithiasis, alcohol abuse/dependence, hypertriglyceridemia, obstruction of the bile duct, and hypercalcemia, SLE was an independent risk factor for higher inpatient mortality (adjusted odds ratio (aOR) 1.88, 95% CI: 1.52-2.33, p < 0.05), respiratory failure (aOR 1.44, 95% CI: 1.24-1.67, p < 0.05), acute renal failure (aOR 1.44, 95% CI: 1.30-1.59, p < 0.05), hypotensive shock (aOR 1.54, 95% CI: 1.34-1.76, p < 0.05), stroke (aOR 2.14, 95% CI: 1.43- 3.21, p < 0.05), and sepsis (aOR 1.33, 95% CI: 1.17-1.51, p < 0.05). Although prevalence of ileus was higher in patients without SLE in Table 2, odds of ileus (aOR 0.94, 95% CI: 0.75-1.16, p = 0.55) was not statistically significant after adjusting for confounding factors.

**Table 3:** Multivariate regression analysis of clinical outcomes *Adjusted for age, sex, race, Elixhauser Comorbidity Index, and etiologies of pancreatitis such as cholelithiasis, alcohol abuse/dependence, hypertriglyceridemia, obstruction of the bile duct, and hypercalcemia; CI, confidence interval

Outcome	Adjusted odds ratio* (95% CI)	p-value
Respiratory failure	1.44 (1.24-1.67)	<0.05
Acute renal failure	1.44 (1.30-1.59)	<0.05
Hypotensive shock	1.54 (1.34-1.76)	<0.05
Ileus	0.94 (0.75-1.16)	0.55
Stroke	2.14 (1.43-3.21)	<0.05
Sepsis	1.33 (1.17-1.51)	<0.05
Inpatient mortality	1.88 (1.52-2.33)	<0.05

## Discussion

Prior to this study, it had been reported that SLE-associated AP carried a mortality rate as high as 27% [8]. However, the data on how coexisting SLE impacts clinical outcomes of patients hospitalized with AP were not well explored. As shown above, these data demonstrate that patients with the diagnosis of SLE, regardless of the etiology of AP, were at higher risk for longer hospital stays, severe complications, and inpatient mortality. Consistent with the aforementioned case report, higher inpatient mortality (aOR 1.88) is seen in AP patients with SLE. In addition, AP patients with SLE are at higher risk for respiratory failure (aOR 1.44), acute renal failure (aOR 1.44), hypotensive shock (aOR 1.54), stroke (aOR 2.14), and sepsis (aOR 1.33). The elevated risk of these renal, pulmonary, and neurologic complications are likely related to the pre-existing disease processes of SLE [13-15]. Many patients with SLE can have a secondary hypercoagulable state, so it is not surprising that among significant adverse outcomes, the aOR for stroke was highest in this study at 2.14. In addition, given that chronic renal disease is a well-known complication of SLE, there is likely an increased susceptibility to an acute or chronic kidney injury, which likely explains the aOR of acute renal failure. Similarly, pulmonary pathologies that can be seen more commonly in SLE patients include interstitial lung disease and pulmonary embolism. Given the increased risk of pulmonary diseases secondary to SLE, this is most likely why this population is at an increased risk for respiratory failure in the setting of acute pancreatitis. SLE patients have dysregulation of the immune system from the disease and immunosuppression and, therefore, are at risk for sepsis and septic shock [16]. This may explain why hypotensive shock and sepsis are seen more in SLE patients with AP compared to non-SLE patients with AP. Likely due to all of these aforementioned reasons, mortality in AP with SLE is higher than in patients who have AP without co-morbid SLE. Given the elevated risk of all of these complications and inpatient mortality, it is not surprising that these patients have a longer hospital stay.

This study suggests that providers should pay particularly close attention to AP patients with SLE during their initial presentation in the emergency department and throughout their hospital course. There are many scoring systems that providers may choose to utilize when making triaging decisions for this population. Some scoring systems that are available include Ranson Criteria, BISAP, PANC3, Modified CT Severity Index, Glasgow-Imrie Criteria, and the Pancreatitis Outcome Prediction score [17-23]. There is no single scoring system that is widely considered superior for predicting inpatient mortality with AP [24]. While not exclusively used for AP, the APACHE II score, in one study, was found to have a high positive predictive value with regards to predicting a fatal outcome secondary to AP as compared to other AP scoring systems [21]. However, none of these scoring systems includes SLE as a factor. Thus, paying particular attention to the presence of comorbid SLE in addition to calculating these scores may assist in providing optimal care to AP patients that may help avoid or mitigate worse outcomes in this patient population.

This study has several limitations. With regards to the increased inpatient mortality, given that there were fewer cases of AP induced by alcohol, cholelithiasis, or hypertriglyceridemia in the SLE group, it is possible that SLE-associated pancreatitis, with its known high mortality, accounted for the overall increased mortality. SLE-associated pancreatitis is difficult to diagnose as it is generally considered a diagnosis of exclusion. Due to the difficulty in diagnosing SLE-associated pancreatitis and that there is no ICD-9 code for SLE-associated pancreatitis, identifying the precise incidence of SLE-associated pancreatitis is difficult to ascertain with the available data. This analysis is also limited by the functionality of performing research with the NIS database. NIS relies on accurate billing code inputs by healthcare providers. Inaccurate billing can result in certain etiologies of AP and the risk for severe complications being over or underrepresented. An additional limitation of NIS is that a timeline of AP severity cannot be tracked. A further study of what proportion of the SLE patient population that presents with only mild to moderate AP and subsequently develops severe complications, as compared to those presenting with already severe AP may provide insight on when and how often an SLE patient with AP should be evaluated given their higher risk profile.

Despite these limitations, one of the greatest strengths of this study is the large sample size, which is able to estimate patient characteristics and outcomes at a national level. In addition, this study was bolstered by the multivariate logistic regression analysis that adjusted for potential confounding factors.

## Conclusions

In summary, AP patients with coexisting SLE are at increased risk for worse outcomes, including higher inpatient mortality and severe complications such as respiratory failure, acute renal failure, hypotensive shock, stroke, and sepsis. Thus, it is essential to critically evaluate how to optimize the care of these patients. Appropriate triaging of care and frequent re-evaluations of the patients by understanding the differences in outcomes of AP between patients with and without SLE may improve outcomes in this patient population.
